# Pilot Study of a Next-Generation Sequencing-Based Targeted Anticancer Therapy in Refractory Solid Tumors at a Korean Institution

**DOI:** 10.1371/journal.pone.0154133

**Published:** 2016-04-22

**Authors:** Hyung Soon Park, Sun Min Lim, Sora Kim, Sangwoo Kim, Hye Ryun Kim, KyuBum Kwack, Min Goo Lee, Joo-Hang Kim, Yong Wha Moon

**Affiliations:** 1 Department of Pharmacology and Brain Korea 21 Plus Project for Medical Sciences, Yonsei University College of Medicine, Seoul, Korea; 2 Division of Medical Oncology, Yonsei Cancer Center, Yonsei University College of Medicine, Seoul, Korea; 3 Medical Oncology, Department of Internal Medicine, CHA Bundang Medical Center, CHA University, Seongnam, Korea; 4 Severance Biomedical Science Institute and Brain Korea 21 Plus Project for Medical Sciences, Yonsei University College of Medicine, Seoul, Korea; 5 Department of Biomedical Science, College of Life Science, CHA University, Seongnam, Korea; National Cancer Center, JAPAN

## Abstract

We evaluated the preliminary efficacy and feasibility of a next-generation sequencing (NGS)-based targeted anticancer therapy in refractory solid tumors at a Korean institution. Thirty-six patients with advanced cancer underwent molecular profiling with NGS with the intent of clinical application of available matched targeted agents. Formalin-fixed paraffin-embedded (FFPE) tumors were sequenced using the Comprehensive Cancer Panel (CCP) or FoundationOne in the Clinical Laboratory Improvement Amendments-certified laboratory in the USA. Response evaluations were performed according to RECIST v1.1. Four specimens did not pass the DNA quality test and 32 specimens were successfully sequenced with CCP (n = 31) and FoundationOne (n = 1). Of the 32 sequenced patients, 10 (31.3%) were ≤40 years. Twelve patients (37.5%) had received ≥3 types of prior systemic therapies. Of 24 patients with actionable mutations, five were given genotype-matched drugs corresponding to actionable mutations: everolimus to *PIK3CA* mutation in parotid carcinosarcoma (partial response) and tracheal squamous cell carcinoma (stable disease; 21% reduction), sorafenib to *PDGFRA* mutation in auditory canal adenocarcinoma (partial response), sorafenib to *BRAF* mutation in microcytic adnexal carcinoma (progressive disease), and afatinib to *ERBB2* mutation in esophageal adenocarcinoma (progressive disease). Nineteen of 24 patients with actionable mutations could not undergo targeted therapy based on genomic testing because of declining performance status (10/24, 41.7%), stable disease with previous treatment (5/24, 20.8%), and lack of access to targeted medication (4/24, 16.7%). NGS-based targeted therapy may be a good option in selected patients with refractory solid tumors. To pursue this strategy in Korea, lack of access to clinical-grade NGS assays and a limited number of genotype-matched targeted medications needs to be addressed and resolved.

## Introduction

In Korea, a total of around 200,000 new solid tumor cases and around 66,000 solid tumor deaths were reported in 2012 [1]. According to solid tumor types, approximately 30% of patients had distant metastasis at the time of cancer diagnosis [2]. Systemic chemotherapy is the standard treatment for these advanced cancer patients. However, many patients have treatment failure after standard therapy. These refractory solid tumor patients have few anti-cancer treatment options. Many of these patients pay high costs for unproven treatments, such as traditional medicines, but still do not have an improved survival in advanced solid tumors [2]. In Korea, the cost of alternative medicine in cancer patients increased from 621 to 1,388 (million US$, per year) during 2000–2010 [3]. Therefore, development of effective therapeutic strategies for refractory solid tumors is a huge unmet medical need.

Currently, molecular-based targeted therapy is a standard approach in selected patients such as non-small cell lung cancer (NSCLC) with *EGFR* mutations in which a very high response rate of approximately 75% is observed [4]. More recently, the strategy of matching targeted drugs to biologically relevant targets using molecular profiling techniques is becoming better established, although many challenges remain [5,6]. A systematic review of phase II clinical trials in advanced/metastatic NSCLC showed that molecular matching of patients' tumors to drugs was independently associated with better outcomes as compared with those of unselected patients [7]. Moreover, in the phase I setting, molecular matching was associated with improved outcomes in multivariate analysis [8].

Despite such advantages, genotype-matched therapy using molecular profiling in advanced cancer faces various obstacles in many countries. For instance, lack of access to clinical-grade next-generation sequencing (NGS) testing and targeted medication is the most common barrier. Therefore, we performed a pilot study to evaluate the preliminary efficacy and clinical feasibility of NGS-based targeted anticancer therapy at a Korean institution.

## Materials and Methods

### Study design

**Fig 1:**
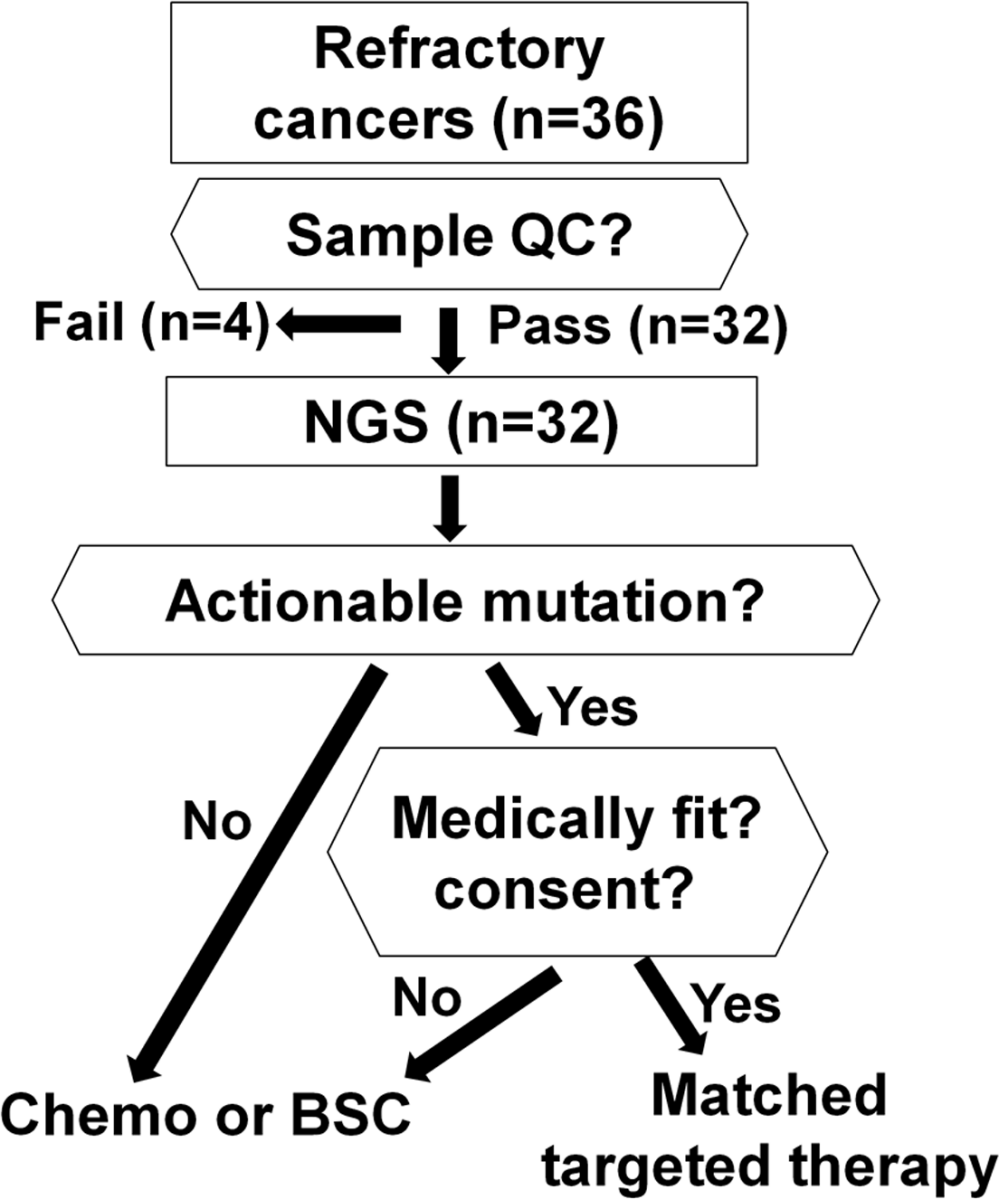
Study scheme. After quality check (QC), samples were sequenced by next generation sequencing (NGS). Medically fit patients with actionable mutation received matched targeted therapy, but patients who were not medically fit received chemotherapy or best supportive care (BSC).

**Fig 1** details the study schematic demonstrating the flow of the patients who consented for the current pilot study of NGS-based targeted anticancer therapy. First, sample quality, such as tissue fragmentation or DNA concentration, was checked using agarose gel or PicoGreen^R^ [9]. After the sample quality check, somatic mutations were identified using FoundationOne by Foundation Medicine or Ion AmpliSeq^TM^ Comprehensive Cancer Panel (CCP, Life Technology) by Macrogen, the Clinical Laboratory Improvement Amendments (CLIA)-certified laboratory, MCL, in Rockville, Maryland [10,11]. In this study, actionable mutation was defined as a mutation that was either the direct target or a pathway component that could be targeted by at least one approved or investigational drug. Patients who harbored actionable mutations were treated using genotype-matched targeted drugs. Approved drugs for the disease or another disease were given on or off-label, respectively. Drugs in clinical trials were considered if available. For instance, everolimus is approved for the treatment of renal cell carcinoma by Korea Food and Drug Administration; however, in this study, everolimus was used in parotid carcinosarcoma and tracheal squamous cell carcinoma with *PIK3CA* mutation. In this study, *TP53* mutations were not considered actionable mutations as in a similar study [8].

### Study objectives

The primary objective of this study was the response rate (RR) as determine by RECIST v1.1 [12] in patients who received genotype-matched therapy. Firstly, radiologists read baseline CT scan, MRI or PET CT, and restaging images were read again after targeted therapy. Then, based on radiologist’ official report, medical oncologists evaluated the tumor response according to RECIST 1.1. Secondary objectives included time to progression (TTP) in the same subset and clinical feasibility of NGS-based targeted anticancer therapy.

### Patient eligibility

From May 2014 to January 2015, patients with advanced cancer underwent molecular profiling with NGS with the intent of clinical application of available matched targeted agents at Severance hospital, Seoul, Korea. Key inclusion criteria were as follows: (1) age ≥ 19 years; (2) refractory solid tumor, which was defined as advanced solid tumor that was refractory to standard therapy and had no more evidence-based therapies; (3) available tumor tissue; (4) Eastern Cooperative Oncology Group (ECOG) performance status of 0–2 at enrollment; (5) appropriate organ functions which allow anti-cancer therapy; and (6) signed informed consent. The study protocol was approved by the Institutional Review Board of Severance Hospital, Yonsei University College of Medicine, Seoul, Korea.

### NGS

Tumor tissue was obtained from biopsy or surgery upon the initial diagnosis of cancer at the primary or metastasis site, and tumor genomic DNA was extracted from formalin-fixed and paraffin-embedded (FFPE) tumor tissue. Extracted tumor genomic DNA was sent to Foundation Medicine or Macrogen, the CLIA-certified laboratory in the USA, and library preparation using FoundationOne or CCP was conducted. CCP is a panel, targeting 409 genes which includes the exons of tumor suppressor genes and oncogenes frequently mutated [13]. The panel was designed to be amplification based capture with approximately 16,000 amplicons. Average amplicon size is 155 base pairs (125–175 base pairs), and it require a total of 40ng of DNA as a template for each sample. Sequencing was processed by Ion PGM^TM^ system. FoundationOne is a pan-cancer panel, which is designed for 315 cancer related genes and 28 frequently rearranged genes [14].

### Analysis platform of NGS data

Our in-house pipeline was applied to analyze CCP data. Sequencing data using CCP was aligned to the human reference genome build 19 and base calling was performed by Ion reporter 4.0 versions (Life Technologies). Variants acquired from the CCP panel were filtered by germline variants acquired from the Korean patients and 1000 genome data including Japanese and Chinese data [15]. We then annotated the variants using ANNOVAR [16], and non-coding regions and synonymous variants were filtered out. Mutations with low depth, which indicate ≤50x depths, were filtered out [17]. In addition, mutations with ≤5% variant allele frequency were filtered out [17]. Quality score, which is one parameter of the variant call format (VCF) using the phred scale, was used to filter out the variants, and Q30 was used for cut-off value [11,17]. Finally, we reviewed the mutation using the Broad’s Integrative Genomics Viewer [18]. Variants acquired from the CCP panel were validated by Sanger sequencing in selected actionable targets. In FoundationOne, a mutation list was provided by the service provider.

## Results

### Patient characteristics

A total of 36 patients were enrolled in the pilot study, and samples from 32 patients passed the quality control test for molecular analysis. Baseline characteristics are presented in **Table 1**. The median age was 48.5 years (range, 22–72 years), and 10 (31.3%) patients were ≤40 years old. Eleven NSCLC (34.4%) patients (10 adenocarcinomas and one neuroendocrine carcinoma) and four (12.4%) esophageal cancer patients were enrolled, and patients with various tumor types were enrolled in small numbers. Types of previous systemic therapy ranged from 1 to 5, and patients with ≥3 previous chemotherapy histories were 12 (37.5%).

**Table 1:** Patient characteristics at time when mutation profiling was performed.

Characteristic		Number (%)
Age, years	Median (Range)	48.5 (22~72)
Sex		
	Male	19 (59.4%)
	Female	13 (40.6%)
ECOG performance status		
	1	17 (53.1%)
	2	14 (43.8%)
	3	1 (3.1%)
Cancer type		
	Non-small cell lung cancer	11 (34.4%)
	Esophageal cancer	4 (12.4%)
	Adenoid cystic carcinoma	2 (6.3%)
	Thymic carcinoma	2 (6.3%)
	Mesothelioma	2 (6.3%)
	Squamous cell cancer of head and neck	2 (6.3%)
	Others^a^	9 (28.1%)
Lines of previous CTx		
	1	13 (40.6%)
	2	7 (21.9%)
	3	3 (9.4%)
	4	6 (18.8%)
	5	3 (9.4%)

ECOG, Eastern Cooperation Oncology Group; CTx, chemotherapy

^a^Others includes 1 external auditory canal adenocarcinoma, 1 breast cancer, 1 parotid carcinosarcoma, 1 endometrial clear cell carcinoma, 1 colon cancer, 1 microcystic adexal cancer of scalp, 1 non-clear renal cell cancer, 1 seminoma, 1 tracheal squamous cell carcinoma.

### NGS test results

**Fig 2:**
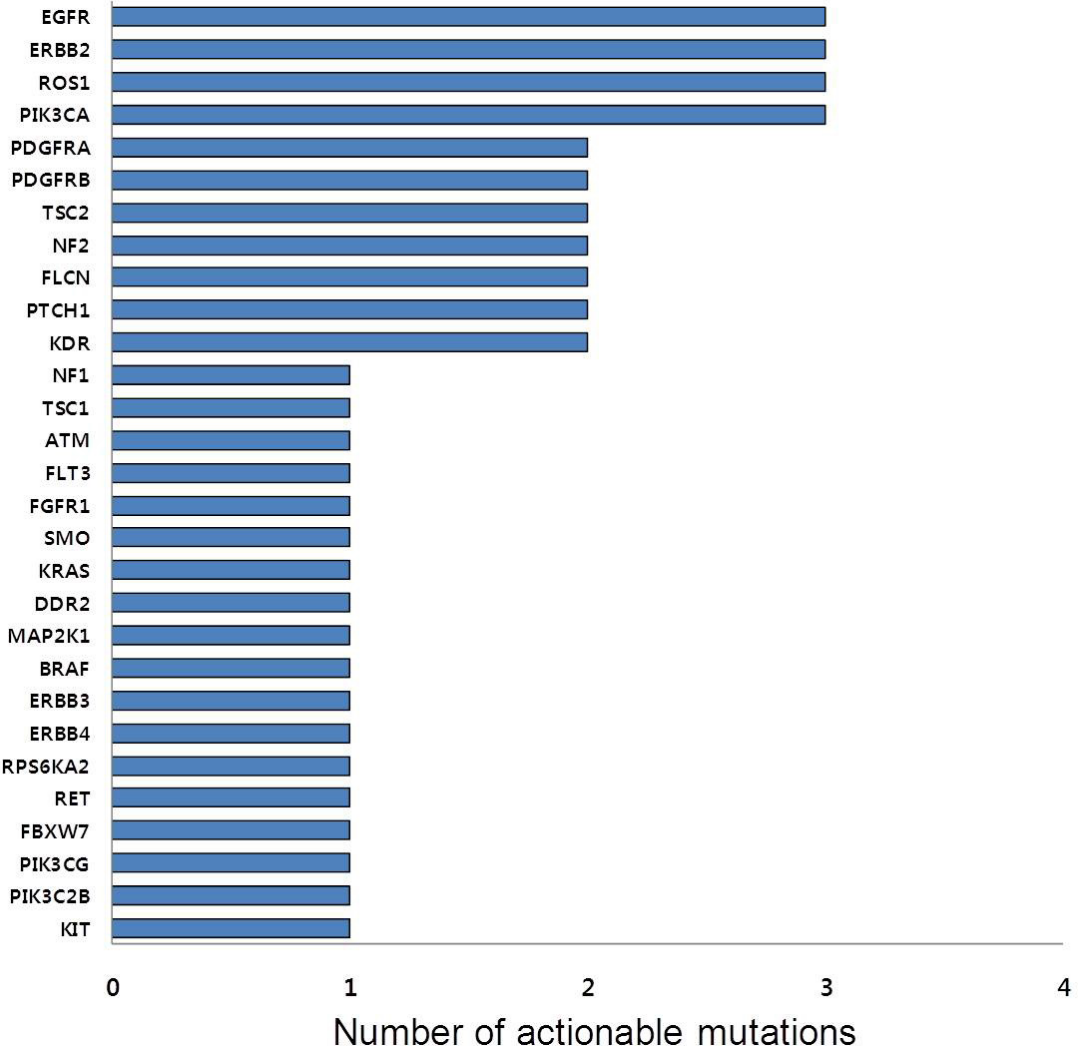
Frequency of each actionable mutation.

To identify somatic mutations, we performed the CCP platform on 31 samples and FoundationOne on one sample to a median depth of 823x and 562x, respectively. We identified 44 actionable mutations which were missense or truncation mutations. The list of actionable mutations in all patients is described in **Table 2**. Twenty-four (75%) of 32 patients had ≥1 actionable mutations; 11 (34.4%), 8 (25%), 3 (9.4%), and two (6.3%) patients had 1, 2, 3, and 4 actionable mutations, respectively. The frequency of each actionable mutation is shown in **Fig 2,** and the most common mutations were *EGFR*, *ERBB2*, *ROS1*, and *PIK3CA* mutations (3/32, 9.4%).

**Table 2:** List of actionable mutations in all patients.

No	Cancer type	Chr	Loci	Ref/Alt	Gene	Mutation type	Platform
1	Adenocarcinoma, lung	22	30079008	G/T	NF2	p.Ser568Ile	CCP
2	Breast cancer	17	29684104	T/C	NF1	p.Val415Ala	CCP
3	Adenocarcinoma, lung	7	55242463	AAGGAATTAAGAG/A	EGFR	Exon 19 del	CCP
3	Adenocarcinoma, lung	7	106508614	C/T	PIK3CG	p.Thr203Met	CCP
3	Adenocarcinoma, lung	9	98242853	C/T	PTCH1	p.Arg104Gln	CCP
4	ADC, external auditory canal	4	55144656	G/C	PDGFRA	p.Leu710Phe	CCP
4	ADC, external auditory canal	4	153244124	G/C	FBXW7	p.Ser598*	CCP
5	Pericardial mesothelioma	17	17129556	C/A	FLCN	p.Gln110His	CCP
7	ESCC	10	43619231	A/G	RET	p.Arg972Gly	CCP
7	ESCC	6	166836748	G/A	RPS6KA2	p.Pro491Leu	CCP
8	Carcinosarcoma, parotid	3	178952085	A/G	PIK3CA	p.His1047Arg	CCP
8	Carcinosarcoma, parotid	6	117683803	A/C	ROS1	p.Leu1115Arg	CCP
8	Carcinosarcoma, parotid	12	56495022	C/T	ERBB3	p.Arg247Cys	CCP
9	Trachea SCC	3	178952085	A/G	PIK3CA	p.His1047Arg	CCP
9	Trachea SCC	15	66737039	C/T	MAP2K1	p.His101Tyr	CCP
10	Colon cancer	1	162731077	G/A	DDR2	p.Ser311Asn	CCP
11	Renal cell ca, non-clear cell	6	117686800	G/A	ROS1	p.Pro973Ser	CCP
12	Adenoid cystic carcinoma	17	37873715	C/A	ERBB2	p.Pro627His	CCP
13	Adenocarcinoma, lung	7	55242463	AAGGAATTAAGAG/A	EGFR	Exon 19 del	CCP
13	Adenocarcinoma, lung	9	98231106	G/A	PTCH1	p.Pro725Leu	CCP
14	ACC, external auditory canal	12	25398284	CC/AC	KRAS	p.Gly12Val	CCP
14	ACC, external auditory canal	2	212293159	G/C	ERBB4	p.Thr898Ser	CCP
14	ACC, external auditory canal	7	128850877	A/T	SMO	p.Lys575Met	CCP
15	Oropharyx cancer	3	178916946	G/C	PIK3CA	p.Lys111Asn	CCP
16	Pleural mesothelioma	22	30038197	A/T	NF2	p.Lys124*	CCP
16	Pleural mesothelioma	5	149509521	C/T	PDGFRB	p.Glu130Lys	CCP
17	NSCLC, large cell neuroendocrine	8	38283724	C/A	FGFR1	p.Val132Leu	CCP
17	NSCLC, large cell neuroendocrine	16	2114297	A/C	TSC2	p.Ile490Leu	CCP
17	NSCLC, large cell neuroendocrine	17	17124799	C/G	FLCN	p.Gly308Ala	CCP
17	NSCLC, large cell neuroendocrine	6	117641053	C/A	ROS1	p.Gly1973Val	CCP
18	Seminoma	13	28623881	G/A	FLT3	p.Pro258Leu	CCP
22	ESCC	4	55961059	G/A	KDR	p.Arg961Trp	CCP
24	Microcytic adenxal carcinoma	7	140453154	T/C	BRAF	p.Asp22Gly	CCP
26	Adenocarcinoma, lung	11	108139269	G/A	ATM	p.Arg183Gln	CCP
28	EADC	1	204419139	G/C	PIK3C2B	p.Cys691Trp	CCP
28	EADC	17	37873715	C/A	ERBB2	p.Pro597His	CCP
29	Adenocarcinoma, lung	17	37873715	C/A	ERBB2	p.Pro627His	CCP
29	Adenocarcinoma, lung	5	149497184	G/A	PDGFRB	p.Ala1045Val	CCP
30	thymic carcinoma	16	2121538	G/T	TSC2	p.Ala623Ser	CCP
30	thymic carcinoma	9	135779171	C/T	TSC1	p.Arg692Gln	CCP
32	Adenocarcinoma, lung				EGFR	L747_P753insS	Foundation
32	Adenocarcinoma, lung				KDR	Amplification	Foundation
32	Adenocarcinoma, lung				KIT	Amplification	Foundation
32	Adenocarcinoma, lung				PDGFRA	Amplification	Foundation

No, number; Chr, chromosome; Ref, reference; Alt, alteration; ADC, adenocarcinoma; ESCC, esophageal squamous cell carcinoma; SCC, squamous cell carcinoma; ACC, adenoid cystic carcinoma; NSCLC, non-small cell lung carcinoma; EADC, esophageal adenocarcinoma; CCP, comprehensive cancer panel.

### Outcome of genotype-matched therapy

Of 24 patients with actionable mutations, five received genotype-matched targeted therapy according to potential biological impact of mutations and availability of drugs in Korea, but 19 could not begin targeted therapy based on genomic tests because of declining performance status (10/24, 41.7%), stable disease with previous treatment (5/24, 20.8%), and lack of access to targeted medication (4/24, 16.7%). In particular, six of 11 NSCLC patients harbored actionable mutations but none were given genotype-matched therapy.

**Fig 3:**
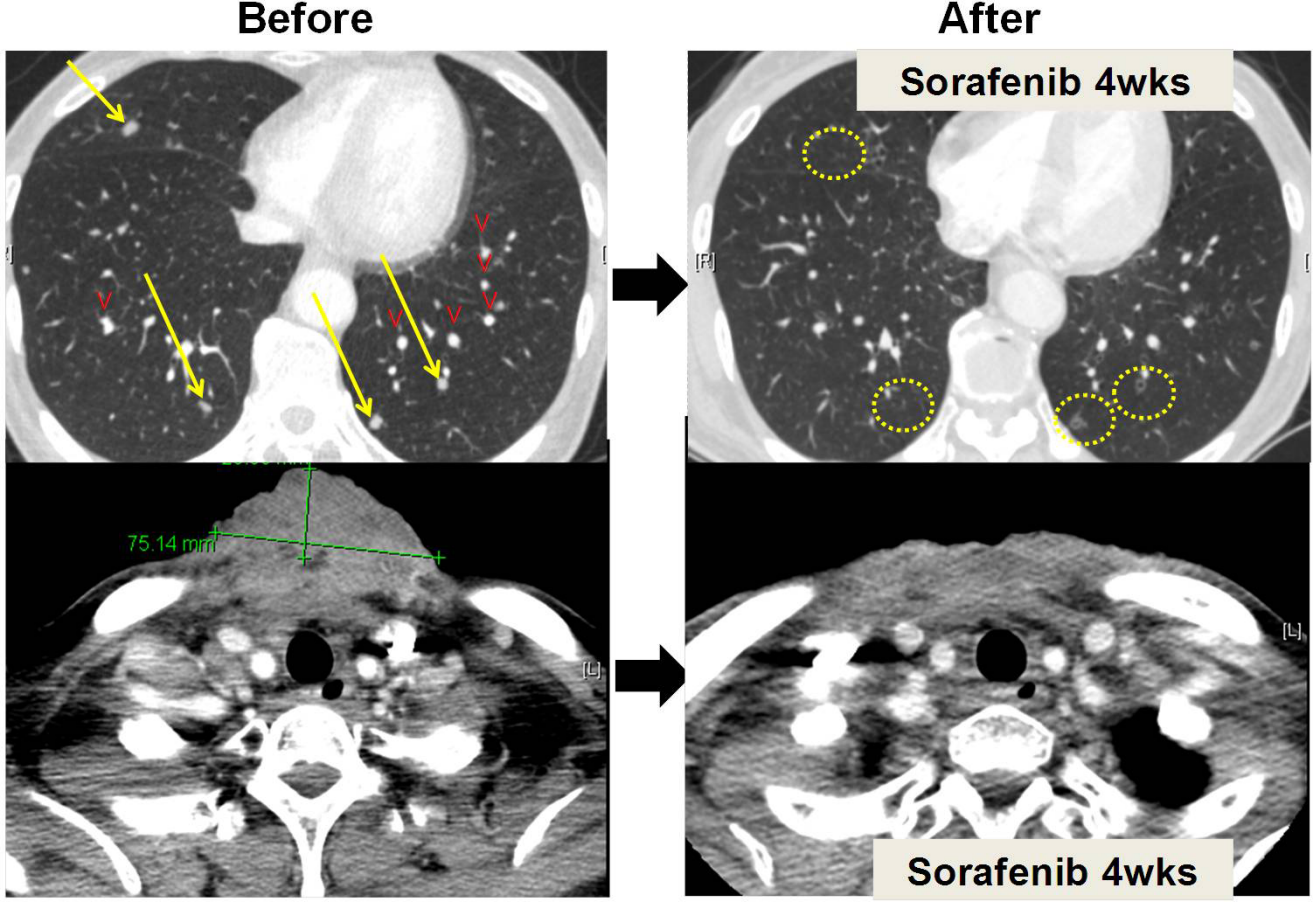
Tumor reduction in external auditory canal adenocarcinoma patient with *PDGFRA* mutation (L710F) after sorafenib treatment. (A and B) multiple lung metastases disappeared and (C and D) huge anterior chest wall mass remarkably shrank after 4 weeks of sorafenib treatment.

Of five patients who received genotype-matched targeted therapy, two showed partial response (PR), one showed stable disease (SD; −21%), but two had progressive disease (PD) just after initiation of matched therapy (**Table 3**). Everolimus was administered to *PI3K/mTOR* pathway genes-mutated patients. A parotid carcinosarcoma patient and tracheal squamous cell carcinoma patient who both had *PIK3CA* mutation (H1047R) showed PR and SD (−21%) to everolimus, respectively. The *PIK3CA* mutation (H1047R) in the parotid carcinoma patient was validated with Sanger sequencing. Before everolimus therapy, both patients received cisplatin based concurrent chemoradiotherapy, and tracheal squamous cell carcinoma patient additionally received gemcitabine and carboplatin chemotherapy, but they became refractory status. Two patients with *PDGFRA* or *BRAF* mutation received sorafenib which is known to target *PDGFR* and *RAF*. The external auditory canal adenocarcinoma patient, who had a *PDGFRA* mutation (L710F), achieved PR with sorafenib (**Fig 3**). Before sorafenib therapy, the patient received several lines of chemotherapy including cisplatin based CCRT, 5-fluorouracil/cisplatin, etoposide/cisplatin, and KX2-391/paclitaxel chemotherapy, but finally progressed to all regimens. However, the microcystic adnexal carcinoma of scalp patient with a *BRAF* mutation (D22G) showed PD to sorafenib within 1 month. Lastly, esophageal adenocarcinoma patient with *ERBB2* mutation (P597H) received afatinib, which targets *ERBB2*, but showed PD within 1 month. Previous treatment history of patients with matched therapy was described in **S1 Table**. TTP in five patients who received genotype-matched therapy was 3.7 months (range, 0.7–6.7).

**Table 3:** Outcomes of matched therapy.

Patient number	Age	ECOG status	Tumor type	Targeted mutation	Other mutation	Drug	Best response	TTP (months)
1	63	2	External auditory canal adenocarcinoma	PDGFRA (L710F)	FBXW7	Sorafenib	PR (-68%)	3.7
2	37	1	Parotid carcinosarcoma	PIK3CA (H1047R)	ROS1, ERBB3, TP53	Everolimus	PR (-30%)	5.8
3	48	1	Tracheal squamous cell carcinoma	PIK3CA (H1047R)	MAP2K1	Everolimus	SD (-21%)	6.7
4	49	3	Microcystic adnexal carcinoma of scalp	BRAF (D22G)	None	Sorafenib	PD	0.7
5	63	2	Esophagus adenocarcinoma	ERBB2 (P597H)	PIK3C2B, TP53	Afatinib	PD	0.9

ECOG, Eastern Cooperation Oncology Group; TTP, time to progression; PR, partial response; SD, stable disease; PD, progressive disease.

## Discussion

For patients with refractory solid tumor, genome-based basket [19], umbrella [20], or phase I clinical trials [8] are drawing attentions. The basket trial allows patients with several tumor types with the same actionable target to be enrolled in trial. The other approach is umbrella trial, which is designed to test the impact of different drugs on different mutations in a single type of cancer, on the basis of molecular profile. However, these studies are only accessible in a limited number of countries. In a Korean institution, we evaluated the preliminary efficacy and clinical feasibility of NGS-based anticancer therapy in refractory solid tumors.

In this study, NGS-based genotype-matched therapy showed efficacy in patients with refractory solid tumors for which we did not have standard therapeutic options, although only small number of patients received genotype-matched therapy. Of five patients with refractory solid tumor who received genotype-matched therapy in this study, significant tumor reduction was seen in three patients (60%; two PRs, one decreasing SD by −21%). In a phase I Program at MD Anderson Cancer Center in the similar clinical setting, the response rate (RR) was 25% [8], whereas RR was only 4% in the patients with non-matched therapy [7]. However, a recently published SHIVA trial [21], which was a randomized, phase II trial to compare genome-based molecularly targeted therapy versus conventional therapy for advanced cancer, failed to show any improvement in survival or responses with genome-based targeted therapies. However, weakness of SHIVA trial can be found [22]: 1) patients with several co-existing molecular alterations are unlikely to respond to a single targeted therapy [23], 2) hormone monotherapy in heavily treated patients is unlikely to bring clinical response [24], 3) some targeted agents were incorrectly matched to molecular alterations [25], 4) treatment in the control group was offered at the discretion of physicians which may lead biases. Furthermore, two meta-analyses in 70,000 patients reported that trials with a personalized strategy led to a higher proportion of patients achieving responses and longer progression-free and overall survival than trials with unselected patients [26,27]. Therefore, it is hard to generally accept the conclusions of the SHIVA trial that precision therapy is disappointing and that the use of targeted drugs off-label should be discouraged. Taken together, although the current study is a small pilot trial in Korea, we suggest that NGS-based genotype-matched therapeutic approaches may be feasibly tested in larger trials and also may provide reasonable treatment options to refractory solid tumor patients in Korea in the future.

Two patients whose tumors responded to everolimus (one PR, one SD with 21% decrement) had the *PIK3CA* mutation (H1047R), which is already known to be a predictive marker for *PI3K/AKT/mTOR* pathway inhibitors [28]. An external auditory canal adenocarcinoma patient who showed PR to sorafenib harbored the *PDGFRA* mutation (L710F). The *PDGFRA* mutation is observed in approximately 7% of gastrointestinal stromal tumor, and 80% of the *PDGFRA* mutations are found in exon 18, which is located in the tyrosine kinase domain [29]. Our patient’s *PDGFRA* mutation (L710F) was located in the tyrosine kinase domain but was a novel mutation. A prediction of the functional effect of this novel mutation was performed by PolyPhen-2 [30] and possibly damaging was expected. Previous studies showed that sorafenib could inhibit the proliferation of cell lines or patient’s tumor with *PDGFRA* mutation [31,32]. The patient with a microcystic adnexal carcinoma of scalp who had a *BRAF* mutation (D22G) showed PD to sorafenib. That may be explained from the fact that the patient had a non-V600E *BRAF* mutation, which was not located in the kinase domain [33]. In addition, the patient’s poor general condition partly accounted for early PD. An esophageal adenocarcinoma patient with an *ERBB2* mutation (P597H) also did not benefit from afatinib. Although the mutation locus was in the extracellular domain and could be related to *ERBB2* activation [34,35], the patient also had a *PIK3C2B* mutation, which may cause PI3K pathway activation and subsequent resistance to afatinib.

In this study, we found several obstacles in terms of the clinical feasibility in performing NGS-based targeted anticancer therapy. First, there were no clinical-grade NGS assays available in Korea. Thus, we had to send samples for identifying patient’s mutation profile to a CLIA-certified laboratory in the USA. This made turn-around time about 4 weeks, which was too long for advanced cancer patients whose remaining survival time was not long. Clinical-grade NGS assays should be developed soon in Korea. Simpler hotspot cancer gene panels used in clinical practice may be sufficient to detect actionable mutations. The cost of NGS is also a challenging issue. It is currently not covered by either national health or private insurance in Korea. Therefore, in the near future, discussion should be started on the coverage of NGS by national health insurance or at least by private insurance once to twice per each cancer patient. Second, limited access to targeted medications was the most common reason that patients could not be treated. Genome-based basket or phase I trials, which can be offered to refractory solid tumor patients, are not enough in Korea. Thus, our patients were administered genotype-matched therapy off-label. Therefore, Korean medical oncologists should make vigorous efforts to quickly launch genome-based trials for refractory solid tumor patients.

This study has a few limitations. A very small number of patients were enrolled for evaluating the efficacy of genome-based matched therapy. We need more robust evidence for better clinical outcomes with NGS-based targeted therapy to recommend this approach in clinical practice. Second, paired normal samples were not sequenced for somatic mutation calling. This may be related to false-positive findings of somatic mutations [36]. To fix this problem, we used germline variants acquired from Korean patients and 1000 genome data to filter out false-positive findings in somatic mutations. Third, the novel mutation as an actionable target lacks evidence of functional changes in the protein, although the novel mutation is a missense mutation. It is difficult to predict the functional consequence of the novel mutation. Only functional *in-vitro* and *in-vivo* studies can identify the role of that mutation. As expected, the survival time of refractory solid tumor patients was short, and we did not have time to validate functional change of somatic mutations before we administered drugs to patients. However, some of actionable mutations are currently under functional validation.

In conclusion, NGS-based targeted therapy may be a good option in selected patients with refractory solid tumors. To pursue this strategy in Korea, lack of access to clinical-grade NGS assay and limited number of genotype-matched targeted medications needs to be addressed and resolved.

## Supporting Information

S1 Table Prior lines of therapies in patients who treated with NGS based targeted therapy. (DOCX)
