# Supplementary material for: Pilot Study of a Next-Generation Sequencing-Based Targeted Anticancer Therapy in Refractory Solid Tumors at a Korean Institution
Source: PLoS One. 2016 Apr 22;11(4):e0154133. doi: 10.1371/journal.pone.0154133 (PMC4841558; doi:10.1371/journal.pone.0154133)
Supplement: S1 Table — (DOCX) [file pone.0154133.s001.docx]

**S1 Table. Prior lines of therapies in patients who treated with NGS based targeted therapy.**

| Patient number | Age | ECOG status | Tumor type | Previous therapy #1 | Previous therapy #2 | Previous therapy #3 | Previous therapy #4 | Previous therapy #5 |
| --- | --- | --- | --- | --- | --- | --- | --- | --- |
| 1 | 63 | 2 | External auditory canal adenocarcinoma | CCRT with Cisplatin | 5FU/  Cisplatin | Clinical trials in other institution | Etoposide/  Cisplatin | HM-KX2-391/Paclitaxel |
| 2 | 37 | 1 | Parotid carcinosarcoma | CCRT with Cisplatin |  |  |  |  |
| 3 | 48 | 1 | Tracheal squamous cell carcinoma | CCRT with Etoposide/  Cisplatin | Gemcitabine/  Carboplatin |  |  |  |
| 4 | 49 | 3 | Microcystic adnexal carcinoma of scalp | 5FU/Cisplatin |  |  |  |  |
| 5 | 63 | 2 | Esophagus adenocarcinoma | 5FU/Cisplatin | Docetaxel | Navelbine/  Cisplatin | Weekly methotrexate |  |

ECOG, Eastern Cooperation Oncology Group; CCRT, Concurrent chemoradiotherapy; 5FU, 5-fluorouracil
